# Novel Variant in Exon 3 of the *BMP4* Gene Resulted in Ectopic Posterior Pituitary, Craniocervical Junction Dysmorphism and Limb Anomaly

**DOI:** 10.1155/2022/8059409

**Published:** 2022-05-19

**Authors:** Valeria Calcaterra, Rossella Lamberti, Claudia Viggiano, Paola Baldassarre, Luigina Spaccini, Rosa Maria Alfano, Giana Izzo, Laura Grazia Valentini, Gianvincenzo Zuccotti

**Affiliations:** ^1^Pediatric and Adolescent Unit, Department of Internal Medicine, University of Pavia, Pavia, Italy; ^2^Department of Pediatrics, Pediatric Unit, “Vittore Buzzi” Children's Hospital, Milan, Italy; ^3^Clinical Genetics Unit, Department of Obstetrics and Gynecology, “V. Buzzi” Children's Hospital, University of Milan, Italy; ^4^Human Pathology, ASST Santi Paolo e Carlo, San Paolo Hospital, Milan, Italy; ^5^Pediatric Radiology and Neuroradiology Unit, “Vittore Buzzi” Children's Hospital, Milan, Italy; ^6^Department of Neurosurgery, Fondazione IRCCS Istituto Neurologico Carlo Besta, Milan, Italy; ^7^Department of Biomedical and Clinical Science “L. Sacco”, University of Milan, Milan, Italy

## Abstract

*Introduction*. Pituitary differentiation involves a large number of transcription factors. In particular, *BMP4* expression is fundamental for pituitary gland commitment from the ventral diencephalon, suppressing Shh expression in Rathke's pouch. Pathogenic variants in *BMP4* are reported in the literature with a broad phenotypic spectrum, including pituitary and brain malformations. *Case Presentation*. A five-year-old girl came to medical attention following a mild cervical trauma with onset of cervical pain. On clinical examination at birth, postaxial polydactyly type B of the left hand was observed and removed at 10 months of age. A cervical radiography was performed, and a suspicion of craniocervical junction malformation was made. A magnetic resonance imaging of the cervical spine was made, showing an ectopic posterior pituitary, associated with dysmorphism of the craniocervical junction. The anthropometric parameters were pubertal Tanner stage 1, weight 16 kg (z-score: −1.09), height 107 cm (z-score: −0.76), and BMI 14 kg/m^2^ (z-score: −0.92). Normal hormonal assessment was detected. Genetic analysis via next generation sequencing showed a novel de novo heterozygous variant (c.277 G > *T*, p.Glu93^*∗*^) in exon 3 of *BMP4*. *Discussion*. We described a novel mutation in *BMP4*, resulting in ectopic posterior pituitary with normal hormonal assessment, associated to craniocervical junction dysmorphism and limb anomaly. It is important to monitor patient's growth and puberty and to screen the onset of symptoms related to the deficiency of one or more anterior as well as posterior pituitary hormones.

## 1. Introduction

The pituitary gland is responsible for the regulation of growth, reproduction, and metabolism. It is formed by three lobes with different embryological origins: the anterior and the intermediate lobes derive from the oral ectoderm (Rathke's pouch), while the posterior lobe derives from the neural ectoderm [1, 2]. The development of the anterior gland leads to the differentiation of cell types which secret different hormones including growth hormone (GH), thyroid-stimulating hormone (TSH), prolactin (PRL), follicle-stimulating hormone (FSH), luteinizing hormone (LH), and adrenocorticotrophic hormone (ACTH). The intermediate lobe comprehends cells which secrete proopiomelanocortin (POMC). The posterior gland or neurohypophysis is constituted by the axonal terminals of neurons from the paraventricular and supraoptic nuclei of the hypothalamus, which secrete oxytocin and vasopressin, respectively [1, 3]. The release of the pituitary hormones depends on the control of the hypothalamic factors, such as thyrotrophin-releasing hormone (TRH), corticotrophin-releasing hormone (CRH), gonadotrophin-releasing hormone (GnRH), growth hormone releasing hormone (GHRH), dopamine, and somatostatin (SS) [1–4].

The signalling mechanism in pituitary morphogenesis involves a lot of transcription factors (*Shh*, *P-OTX/Pit1/2*, *BMP4*, *FGF8*, *HESX1*, *PROP1*, *POU1F1*, *LHX3*, *LHX4*, *PITX1*, *PITX2*, *SOX2*, and *SOX3*) [5–7]. In particular, *BMP4* expression is fundamental in the limbs, heart, facial processes, and mesenchymal cells development. *BMP4* is responsible for pituitary gland commitment from the ventral diencephalon, suppressing Shh expression in Rathke's pouch [8, 9]. Pathogenic variants in *BMP4* are reported in the literature with a broad phenotypic spectrum which includes eye anomalies (exophthalmia, anophthalmia, microphthalmia, and sclerocornea), hands and/or feet postaxial polydactyly, ventriculomegaly, reduction of white matter, hypoplasia of the corpus callosum, some minor abnormalities of the face, delayed psychomotor development, and variable intellectual disability [10, 11].

We described a patient with a novel mutation in *BMP4* in which ectopic posterior pituitary with normal hormonal assessment, craniocervical junction dysmorphism, and limb anomaly are associated.

## 2. Case Presentation

A female patient was born by vaginal delivery at 41 weeks and 4 days gestational age, after normal pregnancy. Birth weight was appropriate for gestational age (3230 g). A transient hypoglycemia was recorded in the perinatal course. The familiar history was silent except for the mother with Klippel-Feil syndrome.

On clinical examination at birth, postaxial polydactyly type B of the left hand was observed. This appendix was removed at 10 months of age. Infant's growth weight and neurological development were normal.

At the age of 5 years, the patient came to medical attention, following a mild cervical trauma (somersault on soft pillows) with onset of cervical pain. For worsening pain, unresponsive to anti-inflammatory therapy, a cervical radiography was performed, and a suspicion of craniocervical junction malformation was made. A neurosurgical evaluation was performed to rule out a medical emergency due to this finding.

**Figure 1:**
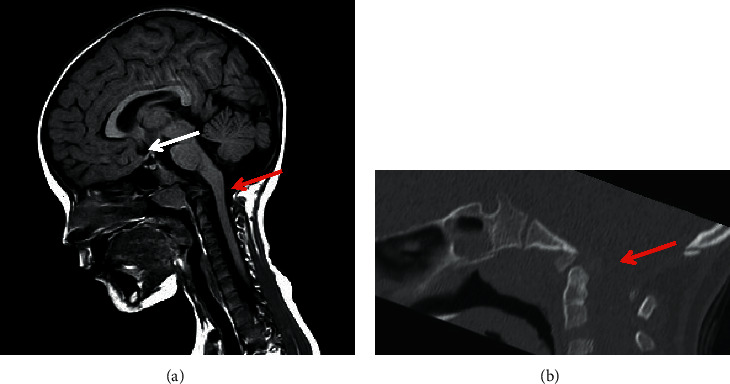
Magnetic resonance sagittal T1 showing the ectopic bright spot of posterior pituitary located along the cranial part of the pituitary stalk (white arrow in Panel (a)). A constitutional dysmorphism of the craniocervical junction characterized by platybasia and basilar footprint with the agenesis of the left posterior hemiarch of the first cervical vertebra (red arrow in Panel (a) and (b)) was also noted.

A magnetic resonance imaging (MRI) of the cervical spine was made, showing an ectopic posterior pituitary, associated with a slight enlargement of the ventricular system with asymmetry of the lateral ventricles and constitutional dysmorphism of the craniocervical junction characterized by platybasia and basilar footprint with the agenesis of the left posterior hemiarch of the first cervical vertebra and the asymmetry of the epistropheus (Figure 1).

An endocrinological check-up was performed. The anthropometric evaluation showed weight 16 kg (WHO z-score: −1.09), height 107 cm (WHO z-score: −0.76), and BMI 14 kg/m^2^ (WHO z-score: −0.92), with a pubertal Tanner stage 1.

As given in Table 1, the hormonal dosages (TSH, FT3, FT4, LH, FSH, PRL, ACTH, cortisol, and IGF1) were in range according to the age. Concordance between skeletal age and chronological age was detected.

Furthermore, genetic analysis was performed. DNA was extracted from peripheral blood of both the proband and the parents (QIAamp, DNA mini kit, Qiagen, Germany). Genomic DNA was enriched for the targeted exome with the TSO (TruSight One, clinica exome) (Illumina, San Diego, CA, USA) kit according to the manufacturer's protocol and sequenced on the Illumina MiSeq platform.

Exome sequencing identified a heterozygous missense variant in *BMP4*: c.277 G > *T*; p.(Glu93^*∗*^) in exon 3 (RefSeq NM_001202.3). The variant has been confirmed with Sanger technology, is de novo, absent in the parents, is not reported in the literature, and is not described in the database of polymorphisms (ExAC, gnomAD). This variant is predicted as damaging by several prediction tools and (SIFT, PolyPhen, MutationTaster, FATHMM, VarSome) classified as pathogenic (PVS1,PM2,PP3) according ACMG guidelines [12].

A long-term endocrinological monitoring was proposed and accepted.

## 3. Discussion

In this case report, we described a novel variant in *BMP4* resulted in ectopic posterior pituitary, craniocervical junction dysmorphism, and limb anomaly.

The bone morphogenetic proteins (BMPs) are members of the transforming growth factor-*β* (TGF-*β*) superfamily. TGF-*β* is a group of cytokines with ubiquitous distribution and several biological functions [13]. Initially described as involved in the bone formation, BMPs play crucial roles in many organ systems [14].

BMP family members induce differentiation of bone lineage cells and regulate cellular division, apoptosis, cellular differentiation, and morphogenesis. In particular, *BMP4* is involved in the process of embryogenesis (mesodermal development, cellular commitment during and after the gastrulation process, and tissue development in the lungs, liver, kidney, urinary system, and teeth) [13, 15, 16].

Moreover, *BMP4* is essential in the initial steps of the development of adenohypophysis. In fact, the onset of pituitary organogenesis is characterized by the restriction of *Shh*, *BMP4*, *FGF8*, and *Wnt5a*, which are expressed in the oral ectoderm from the invaginating Rathke pouch [13, 17].

As described in literature, a homozygous mutation of *BMP4* in mice was lethal, while a heterozygous mutation of this gene caused skeletal abnormalities including polydactyly [9, 18, 19]. It was also described that *BMP4* heterozygous null mice is associated with ocular anterior segment abnormalities [20, 21].

Patients with *BMP4* deletions could manifest ocular anomalies, anterior segment dysgenesis with microcornea, and pituitary and brain malformations [22]. Recently, Jaing et al. [23] reported a novel ocular phenotype, characterized by the pathologic myopia rather than microphthalmia, in heterozygous *BMP4* truncations.

Indeed, pathogenic variants in *BMP4* are reported in the literature with a broad phenotypic spectrum which includes eye anomalies (exophthalmia, anophthalmia, microphthalmia, and sclerocornea), hands and/or feet postaxial polydactyly, ventriculomegaly, reduction of white matter, hypoplasia of the corpus callosum at brain MRI, some minor abnormalities of the face, delayed psychomotor development, and variable intellectual disability [10, 11].

However, different phenotypes among patients with the same *BMP4* mutation suggest complex clinical features caused by *BMP4* dysfunction [24].

In our case report, a novel de novo heterozygous variant was detected (c.277 G > *T*, p.Glu93^*∗*^) in exon 3 of *BMP4*, which confers a clinical disorder characterized by polydactyly type B, ectopy of neurohypophysis and dysmorphism of the craniocervical junction. We classified the variant as pathogenic according to ACMG guidelines that deal with evolutionary conservation of DNA sequences and amino acid; functional studies could be useful to define the protein resulting from posttranslational processing and dimerization of the *BMP4* peptide.

Genetic heterogeneity and variable penetrance of this mutation makes genetic diagnosis difficult considering that posterior pituitary ectopia may not present hormonal disturbance [25]. Additionally, as proposed by Rodriguez-Contreras [26], an oligogenic inheritance may contribute to modify phenotypic expressivity of *BMP4* pathogenic variants.

Once ectopic posterior pituitary has been discovered, from an endocrinological point of view, it is important to monitor patient's growth and puberty and to screen the onset of symptoms related to the deficiency of one or more anterior as well as posterior pituitary hormones.

## Figures and Tables

**Table 1:** Hormonal dosages.

	At endocrinological evaluation	Normal range
TSH (mIU/L)	1.06	0.8–4.70
FT3 (pmol/L)	6.2	3.7–6.8
FT4 (pmol/L)	13.2	10.9–18.0
LH (mIU/mL)	<0.1	0.02–0.3
FSH (mIU/mL)	2.2	1.0–4.2
PRL (ng/mL)	9.4	4.8–23.3
ACTH (pg/mL)	24	5–60
Cortisol (ng/mL)	75	48–195
IGF1 (ng/mL)	95	50–233

## Data Availability

The data used to support this study are available from the corresponding author upon request.
